# Supplementary material for: The psychological distress of parents is associated with reduced linear growth of children: Evidence from a nationwide population survey
Source: PLoS One. 2021 Oct 26;16(10):e0246725. doi: 10.1371/journal.pone.0246725 (PMC8547691; doi:10.1371/journal.pone.0246725)
Supplement: S1 Appendix — (DOCX) [file pone.0246725.s001.docx]

| S1 Appendix. Relative risk ratio of parental distress and other risk factors associated to stunting using multilevel multinomial logistic regression (continued) | | | | | | | | | | | | | | | | | | |
| --- | --- | --- | --- | --- | --- | --- | --- | --- | --- | --- | --- | --- | --- | --- | --- | --- | --- | --- |
| **Risk factors** | **Full Model** | | | | | | | | | **Best Fit Model** | | | | | | | | |
|  | **Mild Stunting** | | | **Moderate Stunting** | | | **Severe Stunting** | | | **Mild Stunting** | | | **Moderate Stunting** | | | **Severe Stunting** | | |
|  | **RRR** | **CI 95%** | **p** | **RRR** | **CI 95%** | **p** | **RRR** | **CI 95%** | **p** | **RRR** | **CI 95%** | **p** | **RRR** | **CI 95%** | **p** | **RRR** | **CI 95%** | **p** |
| Parental distress |  |  |  |  |  |  |  |  |  |  |  |  |  |  |  |  |  |  |
| No distress | 1 |  |  | 1 |  |  | 1 |  |  | 1 |  |  | 1 |  |  | 1 |  |  |
| Maternal distress | 1.31^***^ | [1.15,1.50] | <0.001 | 1.27^**^ | [1.10,1.48] | 0.001 | 0.93 | [0.78,1.10] | 0.397 | 1.33^***^ | [1.17,1.50] | <0.001 | 1.25^***^ | [1.10,1.43] | 0.001 | 0.93 | [0.80,1.09] | 0.374 |
| Paternal distress | 1.40^***^ | [1.19,1.66] | <0.001 | 1.15 | [0.95,1.39] | 0.159 | 1.01 | [0.82,1.24] | 0.933 | 1.37^***^ | [1.18,1.60] | <0.001 | 1.28^**^ | [1.08,1.51] | 0.004 | 1.00 | [0.83,1.20] | 0.965 |
| Parental distress | 1.13 | [0.83,1.55] | 0.434 | 1.38^*^ | [1.00,1.90] | 0.049 | 1.30 | [0.92,1.82] | 0.134 | 1.19 | [0.90,1.57] | 0.228 | 1.40^*^ | [1.06,1.85] | 0.019 | 1.18 | [0.87,1.59] | 0.298 |
| Had infectious diseases |  |  |  |  |  |  |  |  |  |  |  |  |  |  |  |  |  |  |
| No disease | 1 |  |  | 1 |  |  | 1 |  |  | 1 |  |  | 1 |  |  | 1 |  |  |
| >1 disease | 1.01 | [0.95,1.06] | 0.858 | 1.09^**^ | [1.02,1.16] | 0.008 | 1.05 | [0.98,1.12] | 0.189 | 1.01 | [0.96,1.06] | 0.782 | 1.07^*^ | [1.01,1.13] | 0.026 | 1.07^*^ | [1.01,1.14] | 0.033 |
| Sex of the child |  |  |  |  |  |  |  |  |  |  |  |  |  |  |  |  |  |  |
| Girl | 1 |  |  | 1 |  |  | 1 |  |  | 1 |  |  | 1 |  |  | 1 |  |  |
| Boy | 0.98 | [0.93,1.04] | 0.546 | 0.99 | [0.94,1.05] | 0.821 | 1.12^***^ | [1.05,1.19] | <0.001 | 1.00 | [0.95,1.05] | 0.933 | 1.02 | [0.97,1.07] | 0.489 | 1.14^***^ | [1.08,1.20] | <0.001 |
| Maternal BMI |  |  |  |  |  |  |  |  |  |  |  |  |  |  |  |  |  |  |
| Normal weight | 1 |  |  | 1 |  |  | 1 |  |  |  |  |  |  |  |  |  |  |  |
| Underweight | 1.06 | [0.95,1.19] | 0.297 | 1.08 | [0.96,1.23] | 0.211 | 1.10 | [0.96,1.25] | 0.156 |  |  |  |  |  |  |  |  |  |
| Obese | 1.07 | [0.98,1.17] | 0.109 | 0.94 | [0.85,1.04] | 0.214 | 0.75^***^ | [0.67,0.84] | <0.001 |  |  |  |  |  |  |  |  |  |
| Maternal height |  |  |  |  |  |  |  |  |  |  |  |  |  |  |  |  |  |  |
| ≥ 150 cm | 1 |  |  | 1 |  |  | 1 |  |  | 1 |  |  | 1 |  |  | 1 |  |  |
| < 150 cm | 1.53^***^ | [1.45,1.63] | <0.001 | 2.19^***^ | [2.06,2.33] | <0.001 | 1.77^***^ | [1.65,1.89] | <0.001 | 1.49^***^ | [1.41,1.57] | <0.001 | 2.14^***^ | [2.02,2.26] | <0.001 | 1.82^***^ | [1.71,1.93] | <0.001 |
| Paternal height |  |  |  |  |  |  |  |  |  |  |  |  |  |  |  |  |  |  |
| ≥ 155 cm | 1 |  |  | 1 |  |  | 1 |  |  | 1 |  |  | 1 |  |  | 1 |  |  |
| < 155 cm | 1.24^***^ | [1.11,1.39] | <0.001 | 1.72^***^ | [1.53,1.93] | <0.001 | 1.85^***^ | [1.65,2.09] | <0.001 | 1.22^***^ | [1.10,1.35] | <0.001 | 1.62^***^ | [1.47,1.80] | <0.001 | 1.81^***^ | [1.63,2.00] | <0.001 |
| Maternal MUAC |  |  |  |  |  |  |  |  |  |  |  |  |  |  |  |  |  |  |
| ≥ 23.5 cm | 1 |  |  | 1 |  |  | 1 |  |  | 1 |  |  | 1 |  |  | 1 |  |  |
| < 23.5 cm | 1.16^***^ | [1.07,1.26] | <0.001 | 1.18^***^ | [1.08,1.28] | <0.001 | 1.24^***^ | [1.13,1.36] | <0.001 | 1.13^***^ | [1.06,1.21] | <0.001 | 1.23^***^ | [1.14,1.32] | <0.001 | 1.30^***^ | [1.21,1.41] | <0.001 |
| Maternal age |  |  |  |  |  |  |  |  |  |  |  |  |  |  |  |  |  |  |
| <25 years | 1 |  |  | 1 |  |  | 1 |  |  |  |  |  |  |  |  |  |  |  |
| 25-34 years | 1.01 | [0.92,1.11] | 0.765 | 0.95 | [0.86,1.05] | 0.291 | 0.91 | [0.82,1.02] | 0.093 |  |  |  |  |  |  |  |  |  |
| ≥ 35 years | 1.00 | [0.90,1.12] | 0.933 | 0.92 | [0.81,1.04] | 0.165 | 0.91 | [0.80,1.04] | 0.154 |  |  |  |  |  |  |  |  |  |
| Paternal age |  |  |  |  |  |  |  |  |  |  |  |  |  |  |  |  |  |  |
| <30 years | 1 |  |  | 1 |  |  | 1 |  |  |  |  |  |  |  |  |  |  |  |
| 30-39 years | 1.07 | [0.99,1.16] | 0.109 | 1.06 | [0.97,1.17] | 0.184 | 0.98 | [0.89,1.07] | 0.609 |  |  |  |  |  |  |  |  |  |
| ≥ 40 years | 1.08 | [0.98,1.20] | 0.136 | 1.02 | [0.91,1.15] | 0.703 | 0.93 | [0.82,1.04] | 0.213 |  |  |  |  |  |  |  |  |  |
| Iodized salt used |  |  |  |  |  |  |  |  |  |  |  |  |  |  |  |  |  |  |
| Yes | 1 |  |  | 1 |  |  | 1 |  |  | 1 |  |  | 1 |  |  | 1 |  |  |
| No | 1.09^*^ | [1.02,1.17] | 0.011 | 1.15^***^ | [1.06,1.24] | <0.001 | 1.03 | [0.95,1.12] | 0.414 | 1.09^**^ | [1.02,1.16] | 0.008 | 1.14^***^ | [1.06,1.22] | <0.001 | 1.07 | [0.99,1.14] | 0.087 |
| Growth Monitoring attended |  |  |  |  |  |  |  |  |  |  |  |  |  |  |  |  |  |  |
| =6 times | 1 |  |  | 1 |  |  | 1 |  |  |  |  |  |  |  |  |  |  |  |
| < 6 times | 0.99 | [0.94,1.05] | 0.753 | 0.98 | [0.92,1.04] | 0.510 | 1.08^*^ | [1.01,1.16] | 0.018 |  |  |  |  |  |  |  |  |  |
| Used poor water quality |  |  |  |  |  |  |  |  |  |  |  |  |  |  |  |  |  |  |
| No | 1 |  |  | 1 |  |  | 1 |  |  |  |  |  |  |  |  |  |  |  |
| Yes | 1.02 | [0.91,1.15] | 0.686 | 0.98 | [0.86,1.11] | 0.716 | 0.97 | [0.85,1.11] | 0.682 |  |  |  |  |  |  |  |  |  |
| Used unprocessed water |  |  |  |  |  |  |  |  |  |  |  |  |  |  |  |  |  |  |
| Yes | 1 |  |  | 1 |  |  | 1 |  |  |  |  |  |  |  |  |  |  |  |
| No | 0.97 | [0.91,1.03] | 0.308 | 0.98 | [0.92,1.05] | 0.654 | 1.01 | [0.94,1.09] | 0.715 |  |  |  |  |  |  |  |  |  |
| Washed hand |  |  |  |  |  |  |  |  |  |  |  |  |  |  |  |  |  |  |
| Yes | 1 |  |  | 1 |  |  | 1 |  |  |  |  |  |  |  |  |  |  |  |
| No | 1.01 | [0.96,1.07] | 0.658 | 1.00 | [0.94,1.06] | 0.989 | 1.04 | [0.97,1.11] | 0.252 |  |  |  |  |  |  |  |  |  |
| Poor garbage disposal |  |  |  |  |  |  |  |  |  |  |  |  |  |  |  |  |  |  |
| No | 1 |  |  | 1 |  |  | 1 |  |  | 1 |  |  | 1 |  |  | 1 |  |  |
| Yes | 1.06 | [1.00,1.13] | 0.067 | 1.18^***^ | [1.09,1.26] | <0.001 | 1.18^***^ | [1.09,1.28] | <0.001 | 1.06 | [1.00,1.13] | 0.061 | 1.15^***^ | [1.08,1.24] | <0.001 | 1.19^***^ | [1.10,1.28] | <0.001 |
| Used of water waste disposal |  |  |  |  |  |  |  |  |  |  |  |  |  |  |  |  |  |  |
| Improved | 1 |  |  | 1 |  |  | 1 |  |  | 1 |  |  | 1 |  |  | 1 |  |  |
| Unimproved | 1.09^*^ | [1.02,1.17] | 0.016 | 1.10^*^ | [1.02,1.20] | 0.017 | 1.01 | [0.92,1.10] | 0.870 | 1.09^*^ | [1.01,1.16] | 0.022 | 1.10^*^ | [1.01,1.19] | 0.023 | 1.01 | [0.93,1.10] | 0.791 |
| Used of toilet |  |  |  |  |  |  |  |  |  |  |  |  |  |  |  |  |  |  |
| Improved | 1 |  |  | 1 |  |  | 1 |  |  |  |  |  |  |  |  |  |  |  |
| Unimproved | 0.99 | [0.89,1.09] | 0.799 | 0.93 | [0.83,1.04] | 0.184 | 1.00 | [0.89,1.13] | 0.970 |  |  |  |  |  |  |  |  |  |
| Used septic tank for stool |  |  |  |  |  |  |  |  |  |  |  |  |  |  |  |  |  |  |
| Yes | 1 |  |  | 1 |  |  | 1 |  |  |  |  |  |  |  |  |  |  |  |
| No | 0.95 | [0.87,1.03] | 0.231 | 0.97 | [0.88,1.06] | 0.497 | 1.08 | [0.98,1.19] | 0.100 |  |  |  |  |  |  |  |  |  |
| Used solid fuel |  |  |  |  |  |  |  |  |  |  |  |  |  |  |  |  |  |  |
| No | 1 |  |  | 1 |  |  | 1 |  |  |  |  |  |  |  |  |  |  |  |
| Yes | 0.96 | [0.88,1.04] | 0.311 | 0.99 | [0.90,1.08] | 0.780 | 0.98 | [0.89,1.08] | 0.630 |  |  |  |  |  |  |  |  |  |
| Paternal smoking |  |  |  |  |  |  |  |  |  |  |  |  |  |  |  |  |  |  |
| No | 1 |  |  | 1 |  |  | 1 |  |  | 1 |  |  | 1 |  |  | 1 |  |  |
| Yes | 1.03 | [0.97,1.09] | 0.314 | 1.08^*^ | [1.01,1.15] | 0.015 | 1.08^*^ | [1.02,1.16] | 0.016 | 1.04 | [0.99,1.09] | 0.143 | 1.07^*^ | [1.01,1.13] | 0.016 | 1.08^*^ | [1.02,1.15] | 0.012 |
| Wealth Quintile |  |  |  |  |  |  |  |  |  |  |  |  |  |  |  |  |  |  |
| Richest | 1 |  |  | 1 |  |  | 1 |  |  | 1 |  |  | 1 |  |  | 1 |  |  |
| Richer | 1.14^***^ | [1.06,1.22] | 0.001 | 1.15^**^ | [1.05,1.25] | 0.002 | 1.12^*^ | [1.02,1.22] | 0.021 | 1.14^***^ | [1.06,1.23] | <0.001 | 1.16^***^ | [1.06,1.26] | 0.001 | 1.12^*^ | [1.03,1.23] | 0.013 |
| Middle | 1.28^***^ | [1.18,1.40] | <0.001 | 1.42^***^ | [1.29,1.57] | <0.001 | 1.37^***^ | [1.24,1.53] | <0.001 | 1.28^***^ | [1.18,1.38] | <0.001 | 1.44^***^ | [1.32,1.58] | <0.001 | 1.41^***^ | [1.27,1.55] | <0.001 |
| Poorer | 1.23^***^ | [1.10,1.38] | <0.001 | 1.47^***^ | [1.29,1.66] | <0.001 | 1.41^***^ | [1.24,1.61] | <0.001 | 1.22^***^ | [1.12,1.34] | <0.001 | 1.46^***^ | [1.32,1.61] | <0.001 | 1.47^***^ | [1.32,1.63] | <0.001 |
| Poorest | 1.13 | [0.96,1.34] | 0.148 | 1.63^***^ | [1.36,1.95] | <0.001 | 1.62^***^ | [1.34,1.96] | <0.001 | 1.19^***^ | [1.08,1.32] | 0.001 | 1.60^***^ | [1.44,1.79] | <0.001 | 1.77^***^ | [1.57,1.98] | <0.001 |
| Number house member |  |  |  |  |  |  |  |  |  |  |  |  |  |  |  |  |  |  |
| <=4 | 1 |  |  | 1 |  |  | 1 |  |  | 1 |  |  | 1 |  |  | 1 |  |  |
| >4 | 1.06^*^ | [1.00,1.12] | 0.043 | 1.14^***^ | [1.07,1.22] | <0.001 | 1.07^*^ | [1.01,1.15] | 0.034 | 1.05^*^ | [1.00,1.10] | 0.048 | 1.11^***^ | [1.05,1.17] | <0.001 | 1.05 | [0.99,1.11] | 0.112 |
| Maternal education |  |  |  |  |  |  |  |  |  |  |  |  |  |  |  |  |  |  |
| High School | 1 |  |  | 1 |  |  | 1 |  |  | 1 |  |  | 1 |  |  | 1 |  |  |
| Secondary school | 1.13^**^ | [1.05,1.22] | 0.001 | 1.22^***^ | [1.12,1.33] | <0.001 | 1.16^**^ | [1.06,1.27] | 0.001 | 1.12^**^ | [1.05,1.20] | 0.001 | 1.23^***^ | [1.14,1.33] | <0.001 | 1.14^**^ | [1.05,1.24] | 0.001 |
| Primary school | 1.07 | [0.98,1.16] | 0.155 | 1.14^**^ | [1.04,1.26] | 0.007 | 1.14^*^ | [1.03,1.26] | 0.013 | 1.08^*^ | [1.01,1.16] | 0.028 | 1.17^***^ | [1.08,1.27] | <0.001 | 1.12^**^ | [1.04,1.22] | 0.005 |
| No graduation | 1.00 | [0.88,1.13] | 0.948 | 1.10 | [0.96,1.26] | 0.180 | 1.13 | [0.98,1.31] | 0.092 | 0.99 | [0.90,1.09] | 0.856 | 1.14^*^ | [1.03,1.27] | 0.010 | 1.13^*^ | [1.02,1.26] | 0.021 |
| Paternal education |  |  |  |  |  |  |  |  |  |  |  |  |  |  |  |  |  |  |
| > High School | 1 |  |  | 1 |  |  | 1 |  |  |  |  |  |  |  |  |  |  |  |
| Secondary school | 0.99 | [0.92,1.07] | 0.857 | 0.99 | [0.91,1.08] | 0.850 | 0.99 | [0.90,1.08] | 0.800 |  |  |  |  |  |  |  |  |  |
| Primary school | 1.00 | [0.91,1.09] | 0.942 | 1.04 | [0.94,1.14] | 0.451 | 0.99 | [0.90,1.10] | 0.921 |  |  |  |  |  |  |  |  |  |
| No graduation | 1.03 | [0.90,1.17] | 0.668 | 1.08 | [0.94,1.23] | 0.284 | 1.07 | [0.92,1.23] | 0.387 |  |  |  |  |  |  |  |  |  |
| Maternal occupation |  |  |  |  |  |  |  |  |  |  |  |  |  |  |  |  |  |  |
| Office employee | 1 |  |  | 1 |  |  | 1 |  |  | 1 |  |  | 1 |  |  | 1 |  |  |
| Entrepreneurs | 1.31^***^ | [1.16,1.48] | <0.001 | 1.23^**^ | [1.07,1.41] | 0.004 | 1.26^**^ | [1.08,1.46] | 0.003 | 1.29^***^ | [1.14,1.45] | <0.001 | 1.24^**^ | [1.09,1.42] | 0.001 | 1.22^**^ | [1.06,1.42] | 0.007 |
| Farmer | 1.17^*^ | [1.02,1.34] | 0.021 | 1.16 | [1.00,1.34] | 0.051 | 1.38^***^ | [1.18,1.61] | <0.001 | 1.15^*^ | [1.02,1.30] | 0.022 | 1.13 | [0.99,1.29] | 0.072 | 1.31^***^ | [1.14,1.51] | <0.001 |
| Low wages | 1.40^***^ | [1.16,1.70] | <0.001 | 1.19 | [0.97,1.47] | 0.103 | 1.39^**^ | [1.11,1.74] | 0.004 | 1.38^***^ | [1.16,1.65] | <0.001 | 1.17 | [0.96,1.42] | 0.124 | 1.31^*^ | [1.07,1.62] | 0.010 |
| Others | 1.31^***^ | [1.13,1.52] | <0.001 | 1.26^**^ | [1.07,1.49] | 0.006 | 1.21^*^ | [1.01,1.45] | 0.044 | 1.31^***^ | [1.13,1.51] | <0.001 | 1.31^***^ | [1.11,1.53] | 0.001 | 1.12 | [0.94,1.34] | 0.193 |
| Unemployed | 1.24^***^ | [1.14,1.36] | <0.001 | 1.11^*^ | [1.00,1.24] | 0.043 | 1.20^**^ | [1.06,1.34] | 0.003 | 1.24^***^ | [1.13,1.36] | <0.001 | 1.14^*^ | [1.03,1.26] | 0.012 | 1.19^**^ | [1.06,1.33] | 0.002 |
| Maternal occupation |  |  |  |  |  |  |  |  |  |  |  |  |  |  |  |  |  |  |
| Office employee | 1 |  |  | 1 |  |  | 1 |  |  | 1 |  |  | 1 |  |  | 1 |  |  |
| Entrepreneurs | 1.00 | [0.93,1.08] | 0.931 | 1.06 | [0.97,1.16] | 0.215 | 1.14^**^ | [1.04,1.26] | 0.007 | 1.01 | [0.93,1.08] | 0.872 | 1.05 | [0.97,1.15] | 0.242 | 1.15^**^ | [1.05,1.26] | 0.003 |
| Farmer | 1.13^*^ | [1.03,1.25] | 0.011 | 1.17^**^ | [1.06,1.31] | 0.003 | 1.28^***^ | [1.14,1.43] | <0.001 | 1.11^*^ | [1.01,1.21] | 0.023 | 1.14^**^ | [1.04,1.26] | 0.007 | 1.26^***^ | [1.14,1.40] | <0.001 |
| Low wages | 1.11^*^ | [1.00,1.23] | 0.040 | 1.23^***^ | [1.10,1.37] | <0.001 | 1.21^**^ | [1.08,1.37] | 0.002 | 1.10 | [1.00,1.20] | 0.054 | 1.22^***^ | [1.10,1.35] | <0.001 | 1.21^**^ | [1.08,1.35] | 0.001 |
| Others | 1.16^*^ | [1.02,1.32] | 0.021 | 1.08 | [0.93,1.25] | 0.304 | 1.15 | [0.98,1.34] | 0.093 | 1.20^**^ | [1.06,1.36] | 0.004 | 1.11 | [0.96,1.27] | 0.150 | 1.20^*^ | [1.03,1.40] | 0.017 |
| Unemployed | 1.06 | [0.90,1.24] | 0.504 | 0.87 | [0.72,1.05] | 0.157 | 1.13 | [0.93,1.36] | 0.222 | 1.05 | [0.91,1.22] | 0.514 | 0.92 | [0.78,1.10] | 0.370 | 1.19 | [1.00,1.42] | 0.052 |
| Residence |  |  |  |  |  |  |  |  |  |  |  |  |  |  |  |  |  |  |
| Urban | 1 |  |  | 1 |  |  | 1 |  |  | 1 |  |  | 1 |  |  | 1 |  |  |
| Rural | 1.08^*^ | [1.01,1.16] | 0.018 | 1.10^**^ | [1.02,1.18] | 0.010 | 1.15^***^ | [1.06,1.24] | <0.001 | 1.08^*^ | [1.02,1.15] | 0.013 | 1.12^**^ | [1.05,1.20] | 0.001 | 1.16^***^ | [1.08,1.25] | <0.001 |

Exponentiated coefficients; 95% confidence intervals in brackets, aic= LR Test from Aka iki, df_m= degress of freedom of the model, ^*^ *p* < 0.05, ^**^ *p* < 0.01, ^***^ *p* < 0.001
