# Supplementary material for: The psychological distress of parents is associated with reduced linear growth of children: Evidence from a nationwide population survey
Source: PLoS One. 2021 Oct 26;16(10):e0246725. doi: 10.1371/journal.pone.0246725 (PMC8547691; doi:10.1371/journal.pone.0246725)
Supplement: S2 Appendix — (DOCX) [file pone.0246725.s002.docx]

S2 Appendix. HAZ score loss associated with distress of parents and other risk factors

| **Risk factors** | **Best model** |  |  | **Z score** | **%** |
| --- | --- | --- | --- | --- | --- |
|  | **β** | **95% CI** | **p** | **lost** |  |
| **Socioeconomic factors** |  |  |  |  |  |
| Wealth Quintile |  |  |  |  |  |
| Richest | 0 |  |  |  |  |
| Richer | -0.12^***^ | [-0.17,-0.07] | 0.000 | 8064.5 | 12.3 |
| Middle | -0.22^***^ | [-0.27,-0.16] | 0.000 |  |  |
| Poorer | -0.23^***^ | [-0.30,-0.17] | 0.000 |  |  |
| Poorest | -0.34^***^ | [-0.40,-0.27] | 0.000 |  |  |
| Maternal occupation |  |  |  |  |  |
| Office employee | 0 |  |  |  |  |
| Entrepreneurs | -0.15^***^ | [-0.23,-0.07] | 0.000 | 5912.1 | 9.04 |
| Farmer | -0.18^***^ | [-0.26,-0.10] | 0.000 |  |  |
| Low wages | -0.21^***^ | [-0.33,-0.09] | 0.001 |  |  |
| Others | -0.12^*^ | [-0.22,-0.02] | 0.021 |  |  |
| Unemployed | -0.13^***^ | [-0.19,-0.06] | 0.000 |  |  |
| Maternal education |  |  |  |  |  |
| High School | 0 |  |  |  |  |
| Secondary school | -0.084^***^ | [-0.13,-0.04] | 0.001 | 2430.3 | 3.71 |
| Primary school | -0.087^***^ | [-0.14,-0.04] | 0.001 |  |  |
| No graduation | -0.081^*^ | [-0.15,-0.01] | 0.016 |  |  |
| Paternal occupation |  |  |  |  |  |
| Office employee | 0 |  |  |  |  |
| Entrepreneurs | -0.029 | [-0.08,0.02] | 0.279 | 1851.0 | 2.83 |
| Farmer | -0.078^*^ | [-0.14,-0.02] | 0.013 |  |  |
| Low wages | -0.070^*^ | [-0.14,-0.00] | 0.037 |  |  |
| Others | -0.094^*^ | [-0.18,-0.01] | 0.034 |  |  |
| Unemployed | 0.057 | [-0.05,0.16] | 0.285 |  |  |
| Residence |  |  |  |  |  |
| Urban | 0 |  |  |  |  |
| Rural | -0.062^**^ | [-0.11,-0.02] | 0.007 | 1564.9 | 2.39 |
|  |  |  |  | **Group total** | **30.3** |
| **Physiological factors** |  |  |  |  |  |
| Maternal height (cm) |  |  |  |  |  |
| ≥ 150 cm | 0 |  |  |  |  |
| < 150 cm | -0.43^***^ | [-0.46,-0.39] | 0.000 | 6461.5 | 9.88 |
| Sex of the child |  |  |  |  |  |
| Girl | 0 |  |  |  |  |
| Boy | -0.065^***^ | [-0.10,-0.03] | 0.000 | 1517.2 | 2.32 |
| Maternal MUAC (cm) |  |  |  |  |  |
| ≥ 23.5 | 0 |  |  |  |  |
| < 23.5 | -0.14^***^ | [-0.19,-0.09] | 0.000 | 1099.9 | 1.68 |
| Paternal height (cm) |  |  |  |  |  |
| ≥ 155 cm | 0 |  |  |  |  |
| < 155 cm | -0.31^***^ | [-0.38,-0.25] | 0.000 | 1093.4 | 1.67 |
|  |  |  |  | **Group total** | **15.5** |
| **Behavioral factors** |  |  |  |  |  |
| Poor garbage disposal |  |  |  |  |  |
| No | 0 |  |  |  |  |
| Yes | -0.097^***^ | [-0.14,-0.05] | 0.000 | 3302.1 | 5.05 |
| Paternal smoking |  |  |  |  |  |
| No | 0 |  |  |  |  |
| Yes | -0.060^**^ | [-0.09,-0.02] | 0.001 | 1803.5 | 2.76 |
| Iodized salt used |  |  |  |  |  |
| Yes | 0 |  |  |  |  |
| No | -0.075^***^ | [-0.12,-0.03] | 0.001 | 692.3 | 1.06 |
|  |  |  |  | **Group total** | **8.9** |
| **Infectious disease factors** |  |  |  |  |  |
| Had infectious diseases |  |  |  |  |  |
| No disease | 0 |  |  |  |  |
| >1 disease | -0.052^**^ | [-0.09,-0.01] | 0.006 | 740.5 | 1.13 |
|  |  |  |  |  |  |
| **Parental distress** |  |  |  |  |  |
| No distress | 0 |  |  |  |  |
| Maternal distress | -0.086^*^ | [-0.17,-0.00] | 0.048 | 367.3 | 0.56 |
| Paternal distress | -0.11^*^ | [-0.21,-0.00] | 0.047 |  |  |
| Parental distress | -0.19^*^ | [-0.37,-0.01] | 0.045 |  |  |
|  |  |  |  |  |  |
| **Intercept** |  |  |  | 45602.4 | 69.7 |
|  |  |  |  |  |  |
