# Supplementary material for: The psychological distress of parents is associated with reduced linear growth of children: Evidence from a nationwide population survey
Source: PLoS One. 2021 Oct 26;16(10):e0246725. doi: 10.1371/journal.pone.0246725 (PMC8547691; doi:10.1371/journal.pone.0246725)
Supplement: S3 Appendix — (DOCX) [file pone.0246725.s003.docx]

S3 Appendix. Association of distress of parents with other risk factors for stunting

We quantified the proportion of HAZ-score lost in the population using the regression coefficient beta values from Table 2 (presented in Table S2 Appendix) and plotted these against the adjusted relative risk ratio of parental distress associated with these same factors (presented in Table S3 Appendix below). Results are shown in Figure 5a.


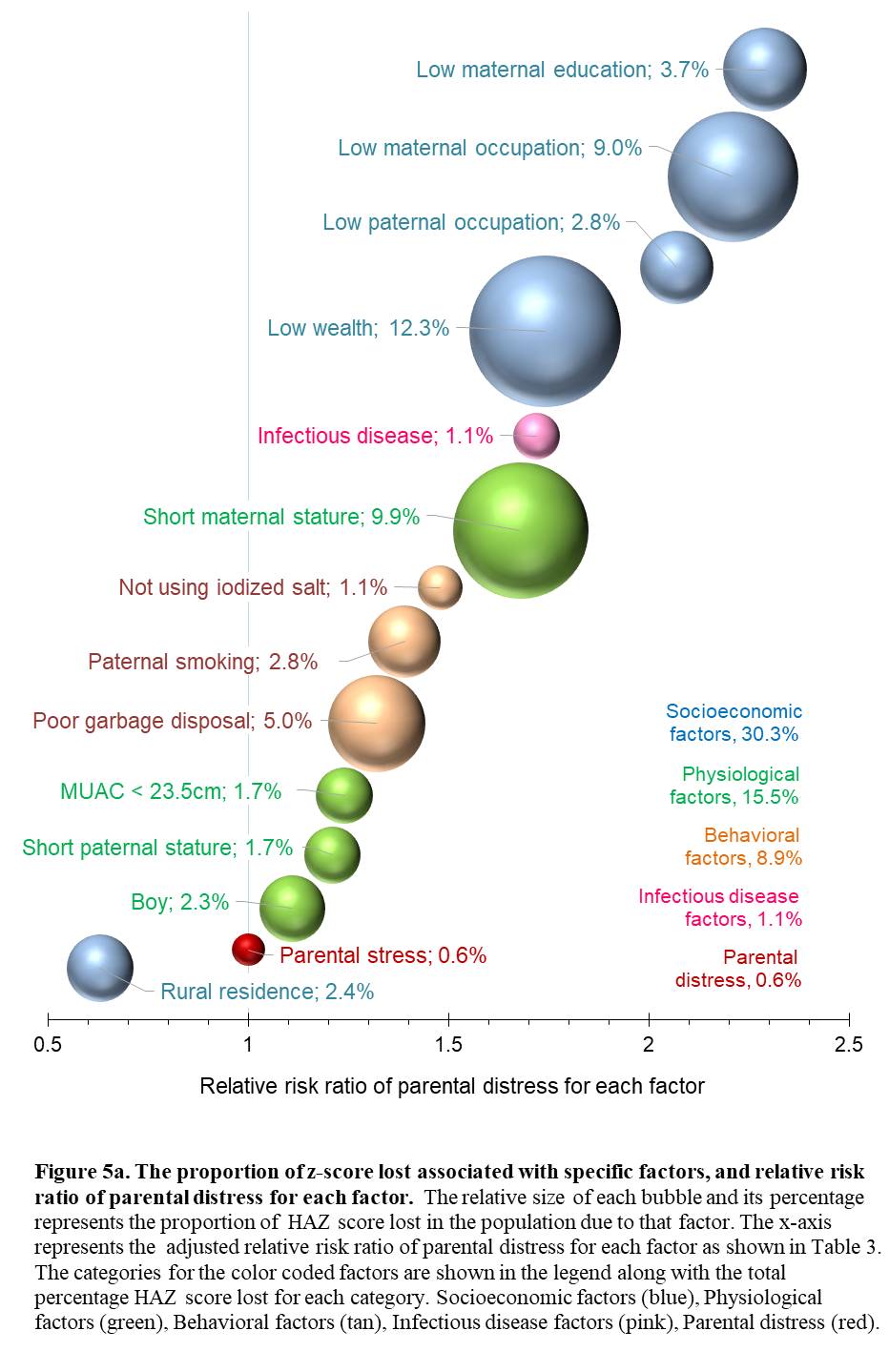


S3 Appendix. Association of distress of parents with other risk factors for stunting

| **Factors** | **Experienced infectious disease** | | | **Not used iodized salt** | | | **Boy** | | | **Low maternal MUAC** | | | **Rural residence** | | |
| --- | --- | --- | --- | --- | --- | --- | --- | --- | --- | --- | --- | --- | --- | --- | --- |
|  | **RR** | **95% CI** | **p** | **RR** | **95% CI** | **p** | **RR** | **95% CI** | p | **RR** | **95% CI** | **p** | **RR** | **95% CI** | **p** |
| Parental distress |  |  |  |  |  |  |  |  |  |  |  |  |  |  |  |
| No distress | 1 | [1,1] |  | 1 | [1,1] |  | 1 | [1,1] |  | 1 | [1,1] |  | 1 | [1,1] |  |
| Paternal distress | 1.24^**^ | [1.08,1.43] | 0.002 | 1.24^*^ | [1.02,1.52] | 0.030 | 0.98 | [0.87,1.10] | 0.741 | 0.96 | [0.81,1.14] | 0.663 | 0.84^*^ | [0.73,0.97] | 0.017 |
| Maternal distress | 1.59^***^ | [1.42,1.77] | 0.000 | 1.24^**^ | [1.06,1.46] | 0.008 | 0.99 | [0.90,1.08] | 0.764 | 1.22^**^ | [1.07,1.39] | 0.003 | 0.76^***^ | [0.68,0.86] | 0.000 |
| Parental distress | 1.72^***^ | [1.37,2.16] | 0.000 | 1.48^*^ | [1.07,2.05] | 0.019 | 1.11 | [0.91,1.35] | 0.296 | 1.24 | [0.94,1.63] | 0.123 | 0.63^***^ | [0.49,0.80] | 0.000 |
|  | **Low Maternal height** | | | **Low Paternal height** | | | **Poor garbage disposal** | | | **Paternal smoking** | | |  | |  |
| Parental distress |  |  |  |  |  |  |  |  |  |  |  |  |  |  |  |
| No distress | 1 | [1,1] |  | 1 | [1,1] |  | 1 | [1,1] |  | 1 | [1,1] |  |  |  |  |
| Paternal distress | 1.38^***^ | [1.22,1.57] | 0.000 | 1.16 | [0.95,1.42] | 0.144 | 0.84 | [0.68,1.05] | 0.132 | 1.14 | [0.99,1.30] | 0.065 |  |  |  |
| Maternal distress | 1.22^***^ | [1.10,1.35] | 0.000 | 1.03 | [0.87,1.22] | 0.737 | 0.85 | [0.72,1.02] | 0.081 | 1.19^**^ | [1.06,1.33] | 0.003 |  |  |  |
| Parental distress | 1.68^***^ | [1.35,2.07] | 0.000 | 1.21 | [0.87,1.67] | 0.251 | 1.32 | [0.88,1.98] | 0.182 | 1.39^**^ | [1.09,1.78] | 0.008 |  |  |  |
| **Wealth Quintile 🡪** |  | **Richer** |  |  | **Middle** |  |  | **Poorer** |  |  | **Poorest** |  |  |  |  |
| Parental distress |  |  |  |  |  |  |  |  |  |  |  |  |  |  |  |
| No distress | 1 | [1,1] |  | 1 | [1,1] |  | 1 | [1,1] |  | 1 | [1,1] |  |  |  |  |
| Paternal distress | 1.36^*^ | [1.07,1.73] | 0.013 | 1.41^**^ | [1.10,1.81] | 0.007 | 1.58^***^ | [1.22,2.05] | 0.000 | 1.87^***^ | [1.43,2.44] | 0.000 |  |  |  |
| Maternal distress | 1.12 | [0.93,1.36] | 0.225 | 1.27^*^ | [1.04,1.54] | 0.017 | 1.50^***^ | [1.23,1.84] | 0.000 | 1.60^***^ | [1.29,1.99] | 0.000 |  |  |  |
| Parental distress | 0.91 | [0.57,1.45] | 0.691 | 1.44 | [0.91,2.26] | 0.116 | 1.5 | [0.94,2.40] | 0.088 | 1.74^*^ | [1.07,2.83] | 0.026 |  |  |  |
| **Maternaloccupation🡪** |  | **Entrepreneurs** |  | **Farmer** | | | **Low wages** | | | **Others** | | | **Unemployed** | | |
| Parental distress |  |  |  |  |  |  |  |  |  |  |  |  |  |  |  |
| No distress | 1 | [1,1] |  | 1 | [1,1] |  | 1 | [1,1] |  | 1 | [1,1] |  | 1 | [1,1] |  |
| Paternal distress | 0.99 | [0.71,1.39] | 0.949 | 1.03 | [0.75,1.42] | 0.848 | 1.07 | [0.70,1.64] | 0.744 | 1.21 | [0.84,1.75] | 0.306 | 1.06 | [0.81,1.39] | 0.672 |
| Maternal distress | 1.38^*^ | [1.05,1.83] | 0.022 | 1.27 | [0.97,1.67] | 0.082 | 1.32 | [0.93,1.86] | 0.120 | 1.38^*^ | [1.01,1.89] | 0.041 | 1.35^*^ | [1.07,1.69] | 0.010 |
| Parental distress | 1.43 | [0.67,3.04] | 0.355 | 2.01^*^ | [1.02,3.98] | 0.044 | 2.21^*^ | [1.01,4.82] | 0.046 | 1.28 | [0.56,2.96] | 0.560 | 1.81 | [0.97,3.38] | 0.063 |
| **Paternal occupation 🡪** |  | **Entrepreneurs** |  |  | **Farmer** |  | **Low wages** | | |  | **Others** |  |  |  |  |
| Parental distress |  |  |  |  |  |  |  |  |  |  |  |  |  |  |  |
| No distress | 1 | [1,1] |  | 1 | [1,1] |  | 1 | [1,1] |  | 1 | [1,1] |  |  |  |  |
| Paternal distress | 1.09 | [0.87,1.36] | 0.459 | 1.08 | [0.85,1.37] | 0.542 | 1.15 | [0.90,1.48] | 0.266 | 1.73^**^ | [1.25,2.41] | 0.346 |  |  |  |
| Maternal distress | 0.87 | [0.73,1.04] | 0.121 | 0.88 | [0.73,1.07] | 0.208 | 1.07 | [0.88,1.29] | 0.492 | 1.08 | [0.81,1.45] | 0.006 |  |  |  |
| Parental distress | 0.84 | [0.55,1.30] | 0.444 | 1.05 | [0.68,1.62] | 0.838 | 1.2 | [0.78,1.85] | 0.399 | 2.07^*^ | [1.19,3.61] | 0.107 |  |  |  |
| **Maternal Education** |  | **Secondary** |  |  | **Primary** |  |  | **No grad** |  |  |  |  |  |  |  |
| Parental distress |  |  |  |  |  |  |  |  |  |  |  |  |  |  |  |
| No distress | 1 | [1,1] |  | 1 | [1,1] |  | 1 | [1,1] |  |  |  |  |  |  |  |
| Paternal distress | 1.31^**^ | [1.09,1.59] | 0.004 | 1.36^**^ | [1.12,1.64] | 0.001 | 1.54^***^ | [1.23,1.93] | 0.000 |  |  |  |  |  |  |
| Maternal distress | 1.34^***^ | [1.15,1.55] | 0.000 | 1.40^***^ | [1.20,1.63] | 0.000 | 1.64^***^ | [1.37,1.96] | 0.000 |  |  |  |  |  |  |
| Parental distress | 1.44^*^ | [1.00,2.05] | 0.049 | 2.00^***^ | [1.42,2.82] | 0.000 | 2.29^***^ | [1.55,3.37] | 0.000 |  |  |  |  |  |  |
|  |  |  |  |  |  |  |  |  |  |  |  |  |  |  |  |
